# Unexpected role of the human cytomegalovirus contribute to essential hypertension in the Kazakh Chinese population of Xinjiang

**DOI:** 10.1042/BSR20171522

**Published:** 2018-06-27

**Authors:** Qian Feng, Jing Hui, Na Tang, Yong-Min Liu, Hua Zhong, Zhen Li, La-Mei Wang, Yuan-Yuan Qu, Feng-Mei Deng, Fang He

**Affiliations:** 1 Department of Pathophysiology/Key Laboratory of Education Ministry of Xinjiang Endemic and Ethnic Diseases, Medical College of Shihezi University, Shihezi, China; 2 Centre of Medical Functional Experiments, Medical College of Shihezi University, Shihezi, China; 3 Department of Respiration Medicine, The First Affiliated Hospital of Medical College of Shihezi University, Shihezi, Xijiang, China; 4 Department of Pathophysiology, Chengdu Medical College, Sichuan, China

**Keywords:** Essential hypertension, Human cytomegalovirus, Inflammatory response, Methylation, Oxidative stress, Renin-angiotensin system

## Abstract

Human cytomegalovirus (HCMV) infection, chronic inflammation and oxidative stress, the renin–angiotensin system (RAS), endothelial function, and DNA methylation play roles in the pathogenesis of essential hypertension (EH); however, the mechanism by which HCMV predisposes patients to hypertension remain unclear. Our group previously demonstrated an association between EH and HCMV infection in Kazakh Chinese. Here, we investigated the relationship between HCMV infection and other clinicopathological features in 720 Kazakh individuals with or without hypertension (*n*=360 each; age: 18–80). Multiple linear and logistic regression analyses were used to determine the associations between HCMV infection, clinical characteristics, and EH. Notably, patients with EH, particularly those with HCMV infection, exhibited a marked increase in tumor necrosis factor-α (TNF-α) and 8-hydroxy-2-deoxyguanosine (8-OHDG) levels, but a decrease in endothelial nitric oxide synthase (eNOS) and renin levels. Similarly, elevated TNF-α and 8-OHDG levels were independent predictors of increased HCMV antibody titers, whereas eNOS and renin were negatively correlated with the latter. Moreover, serum angiotensin-converting enzyme (sACE, *ACE*) methylation was increased, whereas 11-β hydroxysteroid dehydrogenase 2 (HSD11β2; *HSD3B2*) methylation was decreased in patients with EH who were also infected with HCMV. A positive correlation between *HSD3B2* methylation and HCMV IgG titer and blood pressure was additionally observed, whereas angiotensin-converting enzyme (*ACE*) methylation was inversely correlated with blood pressure. Collectively, these data indicate that HCMV may contribute to EH development in the Kazakh Chinese by increasing TNF-α and 8-OHDG levels, suppressing eNOS and renin, and manipulating *HSD3B2* and *ACE* methylation.

## Introduction

Essential hypertension (EH) affects approximately 1 billion individuals worldwide and is a major risk factor for stroke, cardiovascular disease (CVD), and chronic kidney disease [1]. Both genetic and environmental factors are known to facilitate EH development [2,3]; however, the precise pathogenic mechanisms remain unknown, limiting opportunities for early prevention and effective treatment [4].

Human cytomegalovirus (HCMV) is a large dsDNA virus belonging to the β-herpesvirus subfamily [5]. The HCMV infection rate, which ranges from 40 to 100% in adults globally [6], is approximately 90% or higher in China [7]. Notably, our previous study found that HCMV seroprevalence rates of 96.8 and 89.8% in Kazakh and Han patients with hypertension, respectively, are indicative of a link between HCMV infection and EH in these populations [8,9].

HCMV infection is associated with various chronic inflammatory diseases, including CVD, autoimmune diseases, and certain cancers [10]. The role of cytomegalovirus (CMV) in CVD pathogenesis is controversial; however, both HCMV seropositivity and elevated antibody titer are associated with CVD risk [11–14]. Specifically, HCMV infection is known to induce renin and angiotensin II (Ang II) expression in a dose-dependent manner in human vascular endothelial cells and increase arterial blood pressure (BP) by stimulating renin and cytokine production in mice [15].

DNA methylation is a stable epigenetic modification that generally occurs on cytosine residues in cytosine-phosphate-guanine dinucleotides (CpG) in mammalian cells [16]. Emerging evidence suggests that DNA methylation plays an important role in the development of EH [17–20] and several other diseases, including colorectal cancer [21,22], breast cancer [23,24], and coronary artery disease [25]. Aberrant 11-β hydroxysteroid dehydrogenase 2 (*HSD3B2*; HSD11β2) and adrenergic receptor (*AR*) methylation were found to be associated with EH incidence [26] and treatment outcome [27], respectively. Similarly, *α-adducin 1 (ADD1*) promoter methylation (CpG1 and CpG2-5 methylation levels) was inversely correlated with EH risk [28].

Recent studies report that EH is associated with increased HCMV DNA copy number and miRNA expression [29], while HCMV infection alters DNA methylation and induces inflammation *in vivo* [30]. Accordingly, we hypothesized that HCMV infection increases the risk of EH by regulating inflammatory and oxidative responses, the renin–angiotensin system (RAS), endothelial function, and DNA methylation. Hypertension is highly prevalent in the Kazakh Chinese population [31]; thus, we focussed our investigation on a Kazakh community located in Northeastern Xinjiang as the genetic homogeneity and geographic stability of this population, coupled with the shared exposure to common environmental variables, provided a highly favorable opportunity to examine genetic influences on hypertension.

## Materials and methods

### Sample collection

Our study examined 720 subjects of Kazakh descent from rural communities in the Boertonggu countryside of Shawan region in Xinjiang, China from 2009 to 2016: 360 patients with hypertension (167 males and 193 females) and 360 healthy participants (157 males and 203 females) aged 18–80 years. Subjects were selected from a previous case–control study of 2300 participants by random clustering sampling. The study protocol was approved by the ethics committee of Medical College of Shihezi University, and all participants provided written informed consents. All participants completed a detailed questionnaire, underwent a clinical exam, and provided blood samples for measurement of HCMV-specific IgG levels. General details of the Kazakh and Han Chinese populations were obtained from the local government.

Hypertension is defined as systolic blood pressure (SBP) ≥140 mmHg (18.7 kPa) and/or diastolic blood pressure (DBP) ≥90 mmHg (12.0 kPa) according to the 1999 WHO/ISH Hypertension Guidelines. BP was measured three times after the participants rested for 15 min. Other cardiovascular risk factors were also assessed during the examination, including body mass index (BMI), waist-hip ratio (WHR), SBP, DBP, mean arterial pressure (MAP), fasting blood sugar (FBS), total cholesterol (TC), serum low-density lipoprotein cholesterol (LDL-C), serum high-density lipoprotein cholesterol (HDL-C), triglyceride (TG), alcohol consumption, vegetable intake, and smoking habits. Overnight fasting blood (10 ml) was centrifuged, separated, and stored in a freezer at −80°C until analysis. Alternatively, blood cells were collected and their DNA was isolated to measure methylation levels. Subjects lacking lifestyle or clinical data, currently receiving treatment for hypertension, or those with heart, renal, or endocrine diseases known to cause secondary hypertension were excluded from the present study.

### ELISA

Anti-CMV IgG levels were measured using a commercially available ELISA kit (CMV Diagnostic Kit, Haitai Biotech Inc., Zhuhai, China) according to the manufacturer’s instructions. Of the 720 participants, 36 were seronegative (antibody titer <1.1) and 684 were seropositive (≥1.1) based on the assay’s reported sensitivity and specificity (99.9 and 99.8%, respectively).

Plasma levels of C-reactive protein (CRP), tumor necrosis factor-α (TNF-α), NADPH oxidase 4 (NOX4), 8-hydroxy-2-deoxyguanosine (8-OHDG), endothelial nitric oxide (NO) synthase (eNOS), NO, Ang II, and renin were determined with a commercial ELISA kit (Jiancheng Bioengineering Institute, Nanjing, China). Results were read at 450 nm absorbance using a microplate reader (Bio–Rad Laboratories 3550-UV, Carlsbad, CA, U.S.A.).

### Bisulphite sequencing and methylation-specific PCR

Isolated DNA was treated with sodium bisulphite using an EZ DNA Methylation-Gold Kit according to the manufacturer’s instructions with slight modifications. DNA was ethanol-precipitated, dissolved in 50-μl water, and used immediately for PCR under the following conditions: 98°C for 4 min, then 40 cycles of 94°C for 45 s, 64°C for 45 s, 72°C for 1 min, and a final 72°C extension for 8 min. PCR primers, which were designed using Primer Premier 5 software were as follows: angiotensin-converting enzyme *(ACE)* forward, 5′-GATTATGGTTTGGTGAAGAAG-3′; *ACE* reverse, 5′-CTCCTCCRCTCCAAAATC-3′; *HSD3B2* forward, 5′-GGTGTTTTTGTAGGTTTGG-3′; *HSD3B2* reverse, 5′-AAACTTTCCTTCACTTCTCTCC-3′. The recovered PCR products were purified and prepared for TA cloning. Generated plasmids were then examined by sequencing with a 3730xl DNA Analyzer (Applied Biosystems, Foster City, CA, U.S.A.) to analyze methylation status.

### Pyrosequencing

Methylation of top CpG sites in the *ADD1* and sulphatase 1 (*SULF1*) loci was determined by pyrosequencing. After bisulphite treatment, 20 ng converted DNA was used for PCR to amplify the target region. PCR was performed under the following conditions: 95°C for 2 min, then 40 cycles of 95°C for 15 s, 56°C for 15 s, 72°C for 30 s. Primers were as follows: *ADD1* forward, 5′-GTTATGGTTTTAAGGTGGTTAGGTAGA-3′; *ADD1* reverse, 5′-AAAAAACAAAATCCCTAAACTCC-3′; *SULF1* forward, 5′-TGGGGATTTTTAGTGGGATGTATAG-3′, *SULF1* reverse, 5′-TCAATACCAACTACTCCTACATTAAC-3′. The PCR products (10 ml) were sequenced on a Pyrosequencing Q96 HS System (Pyrosequencing, Qiagen) following the manufacturer’s instructions. The methylation status of each locus was analyzed individually as a T/C single nucleotide polymorphism (SNP) using QCpG software (Biotage, Kungsgatan, Sweden).

### Statistical analysis

Data were presented as mean ± S.D. *n* represents the number of independent experiments. For baseline information, the categorical variables were tested using χ^2^ tests and univariate analysis of variance, and *t* test was used for testing the mean differences between various groups. Linear logistic and binary regressions were used to determine the association between CMV infection and laboratory values for BP (as a dependent variable) between subjects with hypertension and control after adjustment for other confounders and stratification. ORs and their 95% CIs were obtained as estimates of associated risk for EH. *P*<0.05 was considered to represent statistical significance. Statistical analyses were performed using SPSS 20.0 software (SPSS Inc., Chicago, IL, U.S.A.).

## Results

### Patient clinicopathological characteristics

A total of 720 Kazakh subjects (360 patients with hypertension and 360 control participants) were randomly selected from a previous case–control study. Clinicopathological findings are shown in Table 1a. As expected, patients with EH displayed common CVD risk factors, including BMI (*P*<0.001), WHR (*P*=0.01), FBS (*P*<0.001), TC (*P*<0.001), TG (*P*<0.001), and LDL-C (*P*<0.001). Alcohol and vegetable intake were significantly higher in subjects with EH than in controls (*P*<0.001). In addition, the EH group showed a marked increase in HCMV seropositivity (*P*=0.04). Table 1b categorizes the patient characteristics according to HCMV infection status. Notably, HCMV infection was more prevalent in younger individuals (*P*=0.011) and females (*P*=0.048) and associated with increased BMI and lower TG levels (both *P*<0.05).

**Table 1a:** Clinicopathological characteristics of 720 Kazakh participants with and without hypertension

Characteristics	Kazakh participants (*n*=720)		*P*-value
	Hypertension	Control	
*n*	360	360	
Males, %	46.4	43.6	0.454
Age (years)	44.69 (11.52)	46.05 (10.13)	0.094
Smokers, % daily	25.3	23.1	0.486
Alcohol, % daily	31.9	16.9	0.000*
Vegetable, % daily	26.4	14.2	0.000*
BMI (kg/m^2^)	27.46 (4.66)	24.40 (4.74)	0.000*
WHR	0.92 (0.41)	0.86 (0.06)	0.01*
SBP (mmHg)	152.05 (22.06)	114.33 (11.87)	0.000*
DBP (mmHg)	99.65 (13.9)	76.08 (7.69)	0.000*
MAP (mmHg)	117.12 (15.17)	88.83 (8.19)	0.000*
FBS (mmol/l)	6.11 (1.50)	5.61 (0.76)	0.000*
TC (mmol/l)	5.11 (1.17)	4.71 (1.08)	0.000*
TG (mmol/l)	1.40 (1.45)	0.83 (0.89)	0.000*
LDL-C (mmol/l)	3.51 (0.85)	3.00 (0.89)	0.000*
HDL-C (mmol/l)	1.42 (0.41)	1.42 (0.29)	0.740
HCMV seropositivity, %	96.7	93.3	0.04*
HCMV IgG antibody titers	3.10 (0.93)	3.13 (1.04)	0.679

Values are expressed as means or percentages (S.D.). Hypertension was defined according to the World Health Organization guidelines. *Statistically significant difference (*P*<0.05).

**Table 1b:** Clinical characteristics of 720 Kazakh study participants with and without serologic evidence of HCMV infection

Characteristics	Kazakh participants (*n*=720)		*P*-value
	HCMV infection	No HCMV infection	
*n*	684	36	
Age (years)	45.32 (10.70)	46.3 (14.10)	0.011*
Males	44.3	61.1	0.048*
Smokers, % daily	23.7	33.3	0.187
Alcohol, % daily	25	11.1	0.058
Vegetable, % daily	79.7	80.6	0.898
BMI (kg/m^2^)	26.05 (4.99)	23.77 (3.09)	0.013*
WHR	0.89 (0.30)	0.86 (0.10)	0.908
Hypertension, %	50.9	33.3	0.04*
SBP (mmHg)	133.27 (25.57)	131.62 (31.41)	0.268
DBP (mmHg)	87.99 (16.19)	85.48 (18.08)	1.57
MAP (mmHg)	103.08 (18.50)	100.86 (21.98)	1.22
FBS (mmol/l)	5.86 (1.21)	5.93 (1.45)	0.290
TC (mmol/l)	4.92 (1.14)	4.57 (1.12)	0.850
TG (mmol/l)	1.10 (1.17)	1.30 (2.10)	0.014*
LDL-C (mmol/l)	3.27 (0.90)	2.91 (0.86)	0.678
HDL-C (mmol/l)	1.42 (0.35)	1.33 (0.44)	0.134

Values are expressed as means or percentages (S.D.). *Statistically significant difference (*P*<0.05).

### Clinical laboratory analysis in hypertensive patients and control subjects

Blood serum analysis revealed that CRP, TNF-α, NOX4, 8-OHDG, and Ang II levels were higher in the EH group than in the controls, whereas eNOS, NO, and renin levels were lower in the former (Table 2; *P*<0.05). The high incidence of HCMV infection in hypertensive participants in our study cohort (96.8%) was found; accordingly, we examined whether the clinical laboratory values differed amongst participants with and without serologic evidence of HCMV infection. We found that levels of CRP, TNF-α, NOX4, 8-OHDG, and Ang II were increased in HCMV seropositive subjects relative to their seronegative counterparts (all *P*<0.05), whereas renin levels were reduced in seropositive individuals (*P*<0.001). Interestingly, eNOS and NO levels have no significance in patients with and without HCMV infection (*P*=0.132 and *P=*0.969).

**Table 2:** Levels of laboratory values in EH and control groups

	EH	Control	*P*-value	HCMV^+^	HCMV^−^	*P*-value
*n*	360	360		684	36	
CRP	17.87 ± 11.64	15.76 ± 10.50	0.011*	17.28 ± 11.22	8.02 ± 1.16	0.000*
TNF-α	313.54 ± 160.92	290.47 ± 146.14	0.044*	308.86 ± 154.98	171.72 ± 17.52	0.000*
NOX4	8.00 ± 4.48	5.64 ± 4.31	0.000*	6.99 ± 4.60	3.51 ± 0.82	0.000*
8-OHDG	22.58 ± 17.25	18.88 ± 16.48	0.003*	21.21 ± 17.26	11.73 ± 2.01	0.001*
eNOS	17.04 ± 2.59	19.14 ± 6.83	0.000*	18.02 ± 5.37	19.38 ± 2.54	0.132
NO	143.90 ± 55.89	154.66 ± 57.96	0.011*	149.30 ± 57.41	148.92 ± 52.77	0.969
Ang II	1524.49 ± 328.25	1450.61 ± 330.79	0.003*	1505.10 ± 328.50	1154.15 ± 171.77	0.000*
Renin	263.03 ± 159.07	937.83 ± 326.23	0.000*	587.23 ± 420.49	851.21 ± 417.82	0.000*

*Statistically significant difference (*P*<0.05).

### Correlation between IgG antibody titer and clinical laboratory values

The independent effects of HCMV infection on EH-associated laboratory values were assessed by multiple linear regression analysis models and adjusted for potential confounders (Table 3). IgG antibody titer was independently correlated to CRP, TNF-α, NOX4, 8-OHDG, and Ang II in all subjects (Table 3; all *P*<0.05). IgG antibody titers were not correlated with those of eNOS, NO, and renin (all *P*>0.05). In hypertensive patients specifically, IgG antibody titers were independently correlated with CRP and 8-OHDG, but negatively correlated with eNOS and Ang II levels (both *P*<0.05). IgG antibody titers were not correlated with TNF-α, NOX4, renin, and NO in hypertension subjects in any of the models (*P*>0.05).

**Table 3:** Linear regression analysis correlations of IgG antibody titers with laboratory values in participants

	All participants (*n*=720)						Hypertension participants (*n*=360)					
	Model 1		Model 2		Model 3		Model 1		Model 2		Model 3	
	B	*P*-value	B	*P*-value	B	*P*-value	B	*P*-value	B	*P*-value	B	*P* value
CRP	1.32	0.002*	1.245	0.004*	1.275	0.003*	1.521	0.021*	1.148	0.09*	1.527	0.024*
TNF-α	16.994	0.003*	12.567	0.032*	12.729	0.030*	5.526	0.544	1.196	0.898	6.897	0.458
NOX4	0.378	0.027*	0.327	0.059	0.310	0.072	0.194	0.445	0.147	0.573	0.079	0.765
8-OHDG	1.880	0.003*	1.753	0.007*	1.728	0.008*	2.473	0.011*	2.494	0.013*	2.289	0.025*
eNOS	−0.08	0.687	−0.079	0.696	−0.071	0.724	−0.396	0.007*	−0.324	0.033*	−0.335	0.030*
NO	0.965	0.655	0.585	0.792	0.715	0.747	0.093	0.977	−1.147	0.728	−0.85	0.799
Ang II	31.001	0.013*	34.818	0.006*	35.512	0.005*	31.001	0.013*	28.269	0.145	31.071	0.115
Renin	−12.351	0.44*	−4.672	0.754	−5.58	0.707	−11.141	0.216	−7.74	0.397	−17.226	0.049

Model 1, unadjusted; Model 2, adjusted for gender, smoking, alcohol consumption, vegetable intake, BMI, WHR, FBS, TC, TG, LDL-C, and HDL-C; Model 3, further adjusted for age. *Statistically significant difference (*P*<0.05).

### Association between HCMV seropositivity and clinical laboratory values

The results from logistic regression analysis between clinical laboratory values and HCMV seropositivity are presented in Table 4a,b. Notably, HCMV seropositivity was associated with increased CRP, TNF-α, NOX4, 8-OHDG, Ang II, and renin before any adjustment (Table 4a; *P*<0.05). Models 2 and 3 show logistic regression analysis performed after adjusting for covariates, which revealed that the association between CRP, TNF-α, NOX4, 8-OHDG, Ang II, renin, and HCMV seropositivity remained significant after controlling for age gender, smoking, alcohol consumption, vegetable intake, BMI, WHR, FBS, TC, TG, HDL-C, LDL-C, and IgG antibody titer (*P*<0.05). In patients with EH specifically, CRP, TNF-α, NOX4, 8-OHDG, eNOS, and Ang II were associated with HCMV infection in all models (Table 4b; *P*<0.05). The association between HCMV seropositivity and renin levels in subject with EH were dampened after adjustment for age, smoking, alcohol consumption, vegetable intake, BMI, WHR, FBS, TC, TG, HDL-C, LDL-C, and IgG antibody titer (OR = 0.995, *P*=0.045 for EH only); however, HCMV seropositivity failed to associate with NO in all models examined (OR = 1, *P*=0.925 for all subjects; OR = 1.001, *P*=0.9 for EH only).

**Table 4a:** Binary logistic regression analysis association of laboratory values with HCMV seropositivity in all participants

(*n*=684)	Model 1		Model 2		Model 3	
	OR (95% CI)	*P*-value	OR (95% CI)	*P*-value	OR (95% CI)	*P*-value
CRP	1.387 (1.236–1.558)	0.000*	1.429 (1.258–1.623)	0.000*	1.437 (1.263–1.635)	0.000*
TNF-α	1.022 (1.014–1.029)	0.000*	1.028 (1.018–1.038)	0.000*	1.028 (1.018–1.038)	0.000*
NOX4	2.03 (1.517–2.715)	0.000*	2.011 (1.436–2.816)	0.000*	2.008 (1.432–2.816)	0.000*
8-OHDG	1.299 (1.167–1.445)	0.000*	1.275 (1.136–1.431)	0.000*	1.271 (1.133–1.426)	0.000*
eNOS	0.976 (0.942–1.011)	0.184	0.973 (0.934–1.013)	0.185	0.974 (0.935–1.014)	0.195
NO	0.969 (0.994–1.006)	1	0.999 (0.993–1.005)	0.773	1 (0.993–1.006)	0.925
Ang II	1.003 (1.002–1.004)	0.000*	1.004 (1.003–1.005)	0.000*	1.004 (1.003–1.006)	0.000*
Renin	0.999 (0.998–0.999)	0.000*	0.999 (0.998–1.000)	0.011*	0.999 (0.998–1.000)	0.007*

Model 1, unadjusted; Model 2, adjusted for gender, smoking, alcohol consumption, vegetable intake, BMI, WHR, FBS, TC, TG, LDL-C, and HDL-C; Model 3, further adjusted for age. *Statistically significant difference (*P*<0.05).

**Table 4b:** Binary logistic regression analysis association of laboratory values with HCMV seropositivity in hypertension participants

(*n*=348)	Model 1		Model 2		Model 3	
	OR (95% CI)	*P*-value	OR (95% CI)	*P*-value	OR (95% CI)	*P*-value
CRP	1.624 (1.224–2.154)	0.001*	1.976 (1.299–3.007)	0.000*	1.956 (1.246–3.072)	0.004*
TNF-α	1.022 (1.010–1.035)	0.000*	1.032 (1.012–1.052)	0.001*	1.048 (1.019–1.078)	0.001*
NOX4	3.536 (1.829–6.838)	0.000*	4.038 (1.731–9.418)	0.001*	3.616 (1.534–8.527)	0.003*
8-OHDG	1.352 (1.136–1.610)	0.001*	1.485 (1.157–1.908)	0.002*	1.569 (1.181–2.084)	0.002*
eNOS	0.661 (0.520–0.839)	0.001*	0.665 (0.491–0.900)	0.008*	0.671 (0.490–0.917)	0.012*
NO	1.002 (0.992–1.013)	0.686	1.001 (0.988–1.014)	0.884	1.001 (0.987–1.015)	0.9
Ang II	1.003 (1.001–1.005)	0.000*	1.004 (1.002–1.007)	0.001*	1.005 (1.002–1.007)	0.002*
Renin	0.996 (0.992–1.000)	0.036*	0.997 (0.993–1.001)	0.184	0.995 (0.990–1.000)	0.045*

Model 1, unadjusted; Model 2, adjusted for gender, smoking, alcohol consumption, vegetable intake, BMI, WHR, FBS, TC, TG, LDL-C, and HDL-C; Model 3, further adjusted for age. *Statistically significant difference (*P*<0.05).

### Correlations between BP and clinical laboratory values

A logistic linear regression analysis revealed that SBP, DBP, and MAP independently correlated with CRP, 8-OHDG, eNOS, Ang II, and renin levels in all models (Table 5a). Moreover, TNF-α correlated with SBP in all models (*P*<0.05), but only associated with MAP (*P*=0.026) before adjustment, and NOX4 correlated with BP before adjustment, but not when adjusted by age, gender, smoking, alcohol consumption, vegetable intake, BMI, WHR, FBS, TC, TG, HDL-C, or LDL-C. In participants with EH specifically, SBP, DBP, and MAP were independently correlated with CRP and TNF-α levels, and negatively correlated with renin in all models (Table 5b; *P*<0.001). Interestingly, Ang II was independently associated with SBP in all models (*P*<0.001), but only associated with MAP (*P*=0.03) before adjustment. Further, a significant correlation was also observed between Ang II and MAP (*P*=0.045) when adjusted by age, gender, smoking, alcohol consumption, vegetable intake, BMI, WHR, FBS, TC, TG, HDL-C, or LDL-C, IgG antibody titers, but not DBP (*P*=0.540). However, BP was not significantly correlated with NO in either EH or all subjects in any model (*P*>0.05).

**Table 5a:** Linear regression analysis correlations of laboratory values with BP in all participants

(*n*=720)		SBP		DBP		MAP	
		B	*P*-value	B	*P*-value	B	*P*-value
CRP	Model 1	1.193	0.000*	0.565	0.000*	0.775	0.000*
	Model 2	1.060	0.000*	0.497	0.000*	0.684	0.000*
	Model 3	1.091	0.000*	0.507	0.000*	0.702	0.000*
TNF-α	Model 1	0.018	0.004*	0.006	0.124	0.010	0.026*
	Model 2	0.015	0.011*	0.004	0.269	0.008	0.069
	Model 3	0.016	0.006*	0.004	0.239	0.008	0.053
NOX4	Model 1	0.574	0.007*	0.412	0.002*	0.466	0.002*
	Model 2	0.318	0.104	0.202	0.111	0.241	0.092
	Model 3	0.346	0.078	0.210	0.098	0.255	0.075
8-OHDG	Model 1	0.227	0.000*	0.120	0.001*	0.156	0.000*
	Model 2	0.184	0.000*	0.089	0.008*	0.184	0.000*
	Model 3	0.196	0.000*	0.093	0.006*	0.127	0.001*
eNOS	Model 1	−1.117	0.000*	−0.613	0.000*	−0.781	0.000*
	Model 2	−0.877	0.000*	−0.462	0.000*	−0.601	0.000*
	Model 3	−0.882	0.000*	−0.464	0.000*	−0.603	0.000*
NO	Model 1	−0.002	0.906	−0.013	0.208	−0.010	0.432
	Model 2	0.003	0.869	−0.010	0.304	−0.006	0.595
	Model 3	0.003	0.851	−0.010	0.309	−0.006	0.607
Ang II	Model 1	0.028	0.000*	0.011	0.000*	0.017	0.000*
	Model 2	0.022	0.000*	0.009	0.000*	0.013	0.000*
	Model 3	0.023	0.000*	0.009	0.000*	0.014	0.000*
Renin	Model 1	−0.05	0.000*	−0.028	0.000*	−0.035	0.000*
	Model 2	−0.047	0.000*	−0.027	0.000*	−0.034	0.000*
	Model 3	−0.048	0.000*	−0.027	0.000*	−0.034	0.000*

Model 1, unadjusted; Model 2, adjusted for age, gender, smoking, alcohol consumption, vegetable intake, BMI, WHR, FBS, TC, TG, LDL-C, and HDL-C; Model 3, further adjusted for IgG antibody titers. *Statistically significant difference (*P*<0.05).

**Table 5b:** Linear regression analysis correlations of laboratory values with BP in hypertension participants

(*n*=360)		SBP		DBP		MAP	
		B	*P*-value	B	*P*-value	B	*P*-value
CRP	Model 1	1.335	0.000*	0.631	0.000*	0.866	0.000*
	Model 2	1.204	0.000*	0.596	0.000*	0.799	0.000*
	Model 3	1.229	0.000*	0.609	0.000*	0.816	0.000*
TNF-α	Model 1	0.060	0.000*	0.021	0.000*	0.034	0.000*
	Model 2	0.047	0.000*	0.018	0.000*	0.027	0.000*
	Model 3	0.047	0.000*	0.018	0.000*	0.027	0.000*
NOX4	Model 1	−0.482	0.063	−0.271	0.098	**−**0.341	0.056
	Model 2	−0.306	0.196	−0.277	0.091	−0.287	0.100
	Model 3	−0.304	0.200	−0.276	0.093	0.285	0.102
8-OHDG	Model 1	0.020	0.773	0.018	0.673	0.018	0.691
	Model 2	0.066	0.282	0.036	0.391	0.046	0.304
	Model 3	0.071	0.248	0.039	0.360	0.050	0.272
eNOS	Model 1	−0.332	0.461	−0.535	0.059	−0.467	0.131
	Model 2	−0.355	0.380	−0.513	0.067	−0.461	0.121
	Model 3	−0.388	0.341	−0.535	0.058	−0.486	0.105
NO	Model 1	−0.016	0.450	−0.005	0.729	0.002	0.877
	Model 2	0.015	0.429	−0.007	0.546	−6.333 × 10^−5^	0.996
	Model 3	0.015	0.434	−0.008	0.560	−1.633 × 10^−4^	0.991
Ang II	Model 1	0.013	0.000*	0.002	0.475	0.005	0.030*
	Model 2	0.011	0.001*	0.001	0.540	0.005	0.051
	Model 3	0.011	0.000*	0.001	0.515	0.005	0.045*
Renin	Model 1	−0.097	0.000*	−0.035	0.000*	−0.055	0.000*
	Model 2	−0.085	0.000*	−0.035	0.000*	−0.051	0.000*
	Model 3	−0.086	0.000*	−0.036	0.000*	−0.051	0.000*

Model 1, unadjusted; Model 2, adjusted for age, gender, smoking, alcohol consumption, vegetable intake, BMI, WHR, FBS, TC, TG, LDL-C, and HDL-C; Model 3, further adjusted for IgG antibody titers. *Statistically significant difference (*P*<0.05).

### Multiple logistic regression analysis of clinical laboratory values and EH

All clinical laboratory values examined were associated with hypertension before adjustment (Table 6; *P*<0.05). Amongst these, when adjusted by age, gender, smoking, alcohol consumption, vegetable intake, BMI, WHR, FBS, TC, TG, HDL-C, or LDL-C, IgG antibody titers, TNF-α, NOX4, 8-OHDG, eNOS, NO, and renin were found to be independent predictors (TNF-α, OR = 1.001, *P*=0.023; NOX4, OR = 1.126, *P*<0.001; 8-OHDG, OR = 1.012, *P*=0.019; eNOS, OR = 0.846, *P*<0.001; NO, OR = 0.996, *P*=0.009; renin, OR = 0.972, *P*<0.001); however, CRP and Ang II showed no independent correlation (CRP, OR = 1.011, *P*=0.178; Ang II, OR = 1, *P*<0.163).

**Table 6:** Binary logistic regression analysis association of laboratory values with hypertension

(*n*=360)	Model 1		Model 2		Model 3	
	OR (95% CI)	*P*-value	OR (95% CI)	*P*-value	OR (95% CI)	*P*-value
CRP	1.017 (1.004–1.031)	0.012*	1.009 (0.994–1.025)	0.232	1.011 (0.995–1.026)	0.178
TNF-α	1.001 (1.000–1.002)	0.045*	1.001 (1.000–1.002)	0.029*	1.001 (1.000–1.002)	0.023*
NOX4	1.136 (1.094–1.180)	0.000*	1.123 (1.078–1.171)	0.000*	1.126 (1.080–1.174)	0.000*
8-OHDG	1.014 (1.004–1.023)	0.004*	1.012 (1.001–1.022)	0.027*	1.012 (1,002–1.023)	0.019*
eNOS	0.827 (0.782–0.874)	0.000*	0.849 (0.798–0.903)	0.000*	0.846 (0.795–0.900)	0.000*
NO	0.997 (0.994–0.999)	0.012*	0.996 (0.993–0.999)	0.009*	0.996 (0.993–0.999)	0.009*
Ang II	1.001 (1.000–1.001)	0.003*	1 (1.000–1.001)	0.219	1 (1.000–1.001)	0.163
Renin	0.976 (0.971–0.981)	0.000*	0.972 (0.965–0.979)	0.000*	0.972 (0.965–0.979)	0.000*

Model 1, unadjusted; Model 2, adjusted for age, gender, smoking, alcohol consumption, vegetable intake, BMI, WHR, FBS, TC, TG, LDL-C, and HDL-C; Model 3, further adjusted for IgG antibody titers. *Statistically significant difference (*P*<0.05).

### DNA methylation analysis

**Figure 1:**
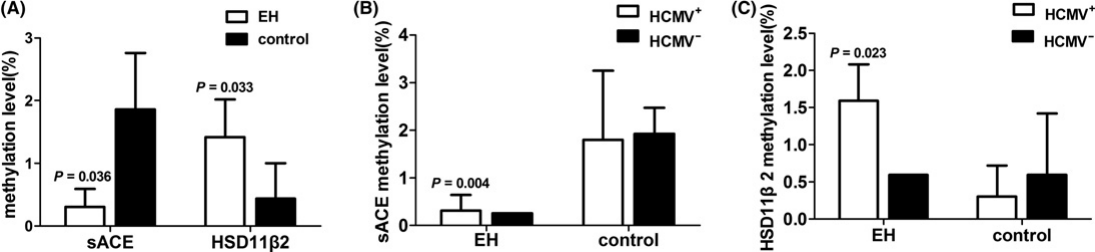
Gene methylation levels in EH (*n*=6) and control groups (*n*=4) (%); HCMV^+^ represents HCMV seropositivity; HCMV^−^ represents HCMV seronegativity (**A**) Serum ACE (*sACE)* and *HSD11β2* gene methylation in the EH group compared with that in the control group (*P*<0.05). (**B**) *sACE* gene methylation in HCMV seropositive subjects relative to HCMV seronegative subjects in the two groups (*P*<0.05). (**C**) *HSD11β2* gene methylation in HCMV seropositive subjects relative to HCMV seronegative subjects in the two groups (*P*<0.05).

Bisulphite sequencing revealed a significant decrease in *ACE* methylation in hypertensive and HCMV seropositivity patients compared with their counterparts (Figure 1; *P*=0.036 and 0.004, respectively). Similarly, *HSD3B2* methylation was higher in subjects with EH than in controls (*P*=0.033) and showed a stronger correlation in those with HCMV seropositivity (*P*=0.023). Unfortunately, no significant difference was observed in *ADD1* and *SULF1* methylation between the EH and control groups (Table 7; *ADD1*: CpG1, *P*=0.581; CpG2, *P*=0.918; CpG3, *P*=0.634; CpG4, *P*=0.878; CpG5, *P*=0.361; SULF1: CpG1, *P*=0.107; CpG2, *P*=0.153).

**Table 7:** Levels of gene methylation in EH and control group

		EH	Control	HCMV infection	No HCMV infection
	*n*	20	10	22	8
ADD1	CpG1	2.80 ± 1.11	3.00 ± 0.81	2.77 ± 1.066	3.13 ± 0.835
	CpG2	2.15 ± 1.27	2.20 ± 1.23	2.09 ± 1.306	2.375 ± 1.061
	CpG3	11.35 ± 3.17	11.80 ± 1.93	11.41 ± 3.081	11.75 ± 2.188
	CpG4	5.15 ± 1.14	5.20 ± 0.63	5.04 ± 1.069	5.62 ± 0.518
	CpG5	7.95 ± 1.82	8.60 ± 1.78	8.14 ± 2.031	8.25 ± 1.035
SULF1	CpG1	15.85 ± 2.70	18.40 ± 4.25	16.877 ± 3.308	16.51 ± 4.036
	CpG2	21.65 ± 3.31	23.80 ± 4.61	22.05 ± 3.946	23.25 ± 3.694

### Correlations between ACE and HSD3B2 methylation, IgG antibody titer, and BP

**Figure 2:**
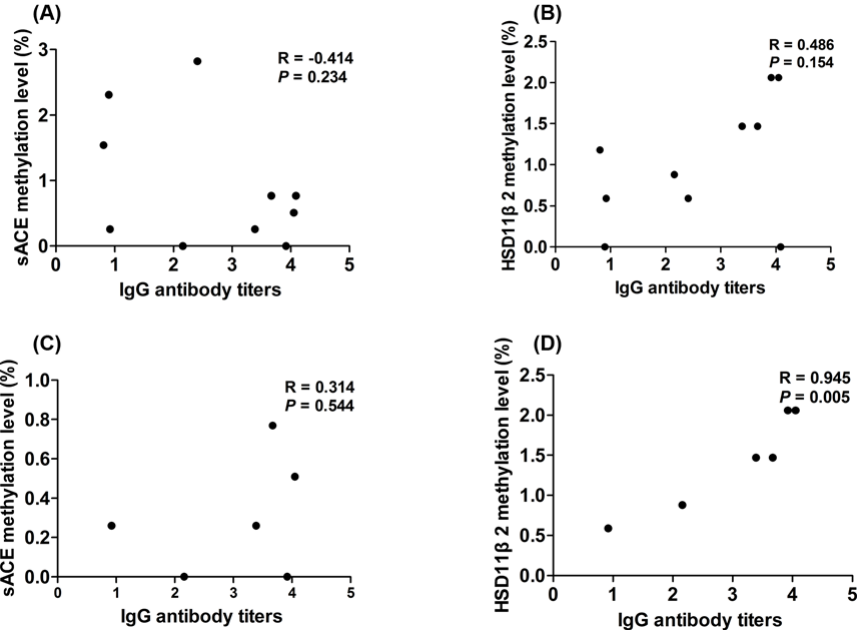
Correlations between IgG antibody titers and gene methylation level in EH and control groups (**A**) Correlation between sACE methylation level and IgG antibody titers in all participants (*n*=10). (**B**) Correlation between HSD11β2 methylation level and IgG antibody titers in all participants (*n*=10). (**C**) Correlation between sACE methylation level and IgG antibody titers in the EH group (*n*=6). (**D**) Correlation between HSD11β2 methylation level and IgG antibody titers in the EH group (*n*=6).

**Figure 3:**
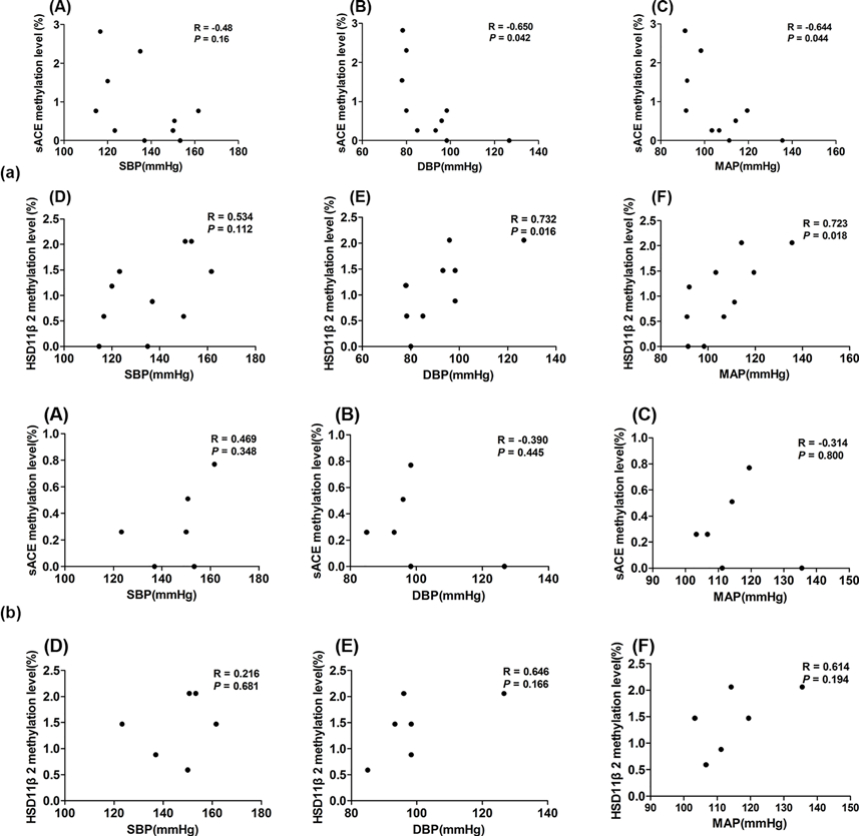
Correlations between gene methylation level and BP in participants (**a**) Correlations between gene methylation level and BP in all participants (*n*=10). sACE, angiotensin converting enzyme; HSD11β2, 11-β hydroxy steroid dehydrogenase 2. (**A**) Correlation between sACE methylation level and SBP in all participants. (**B**) Correlation between sACE methylation level and DBP in all participants. (**C**) Correlation between sACE methylation level and MAP in all participants. (**D**) Correlation between HSD11β2 methylation level and SBP in all participants. (**E**) Correlation between HSD11β2 methylation level and DBP in all participants. (**F**) Correlation between HSD11β2 methylation level and MAP in all participants. (**b**) Correlation between gene methylation levels and BP in the EH group (*n*=6). sACE, angiotensin converting enzyme; HSD11β2, 11-β hydroxy steroid dehydrogenase 2. (**A**) Correlation between sACE methylation level and SBP in the EH group. (**B**) Correlation between sACE methylation level and DBP in the EH group. (**C**) Correlation between sACE methylation level and MAP in the EH group. (**D**) Correlation between HSD11β2 methylation level and SBP in the EH group. (**E**) Correlation between HSD11β2 methylation level and DBP in the EH group. (**F**) Correlation between HSD11β2 methylation level and MAP in the EH group.

We additionally performed linear multiple regression analysis to determine if gene methylation was associated with IgG titer and/or BP. While no correlation was observed between IgG titer and methylation at either locus when analyzing all subjects (Figure 2; *ACE, P*=0.234; *HSD3B2, P*=0.154), a significant correlation was observed between IgG titer and *HSD3B2* methylation in subjects with EH specifically (*P*=0.005). Moreover, we failed to observe an association between gene methylation and SBP (Figure 3a(A,D); *P*=0.160 and *P*=0.112). However, negative correlation was found between *ACE* methylation and both DBP and MAP (Figure 3a(B,C); *P*=0.042, *P*=0.044), while *HSD3B2* was positively correlated with these measures (Figure 3a(E,F); *P*=0.016, *P*=0.018). Further, no correlation between BP and methylation was found at either locus (Figure 3b).

## Discussion

The present study supports that HCMV infection plays an important role in EH pathogenesis through increased TNF-α and 8-OHDG levels, and decreased eNOS and renin levels. Mechanistically, these data suggest that inflammation, oxidative stress, RAS activity, and endothelial dysfunction associated with HCMV infection may enhance susceptibility toward EH. Moreover, HCMV infection results in decreased *ACE* and increased *HSD3B2* methylation. HCMV may aggravate BP through several mechanisms, including activation of RAS, generation of oxidative stress, induction of the inflammatory response, disruption of the endothelial function, and changes in gene expression due to epigenetic modification. Ultimately, these effects may be independently or co-operatively responsible for the elevated BP observed in hypertensive patients.

Our previous study demonstrated that HCMV infection is associated with EH in Kazakh males and Hans in Xinjiang [8]. A sero-epidemiological survey showed that HCMV infection rates in Kazakh and Han adults were 95.4 and 90.1% [8], indicating that HCMV infection may be a predisposing factor for EH [9]. In this setting, increased HCMV sensitivity was also associated with an elevated risk of myocardial infarction and cardiovascular death [32,33]. Since the pathophysiology of atherosclerosis involves inflammation, the possibility of a causal relationship with the atherogenic risk factor hypertension may be assumed. To date, the pathological and molecular mechanisms by which elevated BP leads to vascular disease remain unclear. There is some experimental evidences that hypertension may be associated with HCMV infection; however, to our knowledge, there are no reports describing the mechanisms by which active HCMV infection induces hypertension in the Kazakh Chinese population. This is the first time to explore the complexity of mechanism of EH with HCMV infection in multiple aspects.

Several studies support the participation of inflammatory cytokines in the pathogenesis of hypertension in humans [34,35] and animal models [36,37]. For instance, the acute phase protein CRP is a well-characterized marker of inflammation and tissue damage. Furthermore, elevated circulating CRP levels are an independent risk factor for CVD, including myocardial infarction, stroke, and atherosclerosis [38]. In addition to CRP, the proinflammatory cytokines TNF and interleukin-6 (IL-6) are secreted in response to HCMV infection and are independently associated with mortality due to CVD [38–41]. Hypertensive subjects display increase in serum TNF-α levels and increased arterial stiffness [42]. Interestingly, human aortic smooth muscle cells co-treated with IL-17 and TNF-α exhibit increased vascular fibrosis, cytokine production, and smooth muscle cell proliferation, and subsequently, increase in arterial stiffness and EH risk [43]. Furthermore, some studies have found that TNF-α inhibition reduces SBP and left ventricular hypertrophy via AKT/eNOS pathway activation, thereby improving vascular function in hypertensive rats [44]. Consistently, we found increased CRP and TNF-α levels in HCMV-infected patients with EH, wherein TNF-α was independently associated with EH after adjustment for covariates. Thus, these data suggest that active HCMV infection increases TNF-α secretion, which is subsequently involved in EH pathogenesis.

Landmesser et al. [45] reported that HCMV infection may result in increased levels of oxidative stress and inflammation, which have been linked to endothelial damage in EH [46]. NOX4, as a major source of reactive oxygen species (ROS), is considered to play a crucial role under pathological conditions. The levels of 8-OHDG, which is a marker of ROS-induced DNA damage, are consistent with those of NOX4 and p22 phox protein expression. In addition, ROS increases collagen secretion by vascular smooth cells [47], which may promote vascular stiffness. Accordingly, our result demonstrated that NOX4 and 8-OHDG levels were significantly increased in hypertensive subjects, simultaneously in those with HCMV infection. Moreover, multiple logistic regression analyses showed that NOX4 and 8-OHDG were independently associated with EH; however, multiple linear regression analyses showed an independent positive correlation between 8-OHDG and HCMV antibody titers in all subjects after adjustment for covariates. Therefore, HCMV infection may facilitate 8-OHDG production, oxidative stress, and/or inflammatory response resulting in vascular injury and subsequently EH.

Vascular injury is a frequent characteristic induced by hypertension. Recent evidence indicates that HCMV infection impairs eNOS function and causes endothelial damage [48]. In an animal study, CMV infection was shown to reduce NO bioavailability in endothelial cells and activate NADPH oxidase, resulting in arteriolar dysfunction [49]. Alternatively, CMV may activate the p38-mitogen-activated protein kinase signaling pathway, which may lead to eNOS inhibition, reduced NO production, and subsequent endothelial dysfunction [50–52]. Our results indicate that HCMV-infected patients with EH display lower eNOS levels, while logistic regression analyses suggest that both eNOS and NO reduce the risk of EH. However, only eNOS was shown to reduce the risk of HCMV infection in hypertensive subjects. Further, eNOS level and HCMV antibody titer exhibited an independent negative correlation in hypertensive subjects, suggesting that HCMV mediates eNOS production to induce vascular injury and hypertension.

The observed effects of HCMV infection on the RAS may be responsible for its association with hypertension [53]. An *in vivo* experimental study showed that CMV infection increased arterial pressure, and stimulated renin expression in a dose-dependent manner in kidney cells and vascular endothelial cells, as well as increased Ang II levels in blood and arterial tissues [15]. Ang II stimulates NADPH oxidase activity, increases ROS production, and reduces NO availability leading to endothelial dysfunction [54,55]; however, our results suggest that higher Ang II exression was not an independent risk factor for EH in HCMV-infected patients after adjusting for covariates and IgG antibody titers. Surprisingly, we found that HCMV infection significantly reduced renin-mediated EH incidence and independent negative correlations were observed between renin and HCMV antibody titer and BP in hypertensive subjects. Based on renin expression, EH may be divided into three types – high, normal, and low. Approximately 25–33% of hypertensive patients exhibit low renin levels. However, in China, low-renin hypertension (LRH) accounts for a large roportion of cases of primary hypertension; at present, it has been widely accepted as a subtype of hypertension [18].

Mechanistically, emerging evidence from animal studies suggest a key role for DNA methylation in EH pathogenesis [17,19,56,57]. Zill et al. [58] demonstrated that *ACE* CpG methylation is a pathogenic factor in major depression and CVD, and appears to influence sACE protein expression and inflammatory marker expression in the latter. Similarly, *HSD3B2* promoter methylation highlights a potential mechanism by which HSD11β2 facilitates the pathogenesis of EH and glucocorticoid-induced hypertension. These results strongly support that even mild changes in the HSD11β2 activity due to aberrant site-specific methylation may affect BP regulation [59–62]. Friso et al. [26] also demonstrated that HSD11β2 promoter methylation is associated with decreased expression and protein activity and EH risk in glucocorticoid-treated patients. Consistently, we found that *ACE* methylation was lower in the hypertension group than in controls, whereas *HSD3B2* methylation was higher in subjects with EH independent of HCMV infection. Moreover, MAP and DBP were negatively associated with *ACE* methylation in all subjects. ACE is a membrane-bound endopeptidase involved in angiotensin I and bradykinin metabolism, which are important for vascular tone and cardiac function [63]. We also discovered that the HCMV antibody titer, MAP, and DBP were significantly correlated with *HSD3B2* methylation in EH. The loss of HSD11β2 activity leads to cortisol-induced mineralocorticoid receptor activation, resulting in renal sodium retention [64] and increased BP [65,66]. Therefore, decreased *ACE* and elevated *HSD3B2* methylation likely play crucial role in the development of EH.

*ADD1* methylation has been previously shown to represent a risk factor for EH in males (CpG2-5) and females (CpG1) [28], wherein lower *ADD1* methylation causes higher protein expression and increased Na^+^–K^+^ pump activity, resulting in Na^+^ reabsorption and hypertension. Similarly, Xiaoling Wang et al. observed increased methylation levels at two CpG sites in *SULF1* in EH cases via genome-wide methylation analysis [67]. Accordingly, we hypothesized that *ADD1* and *SULF1* methylation were related to HCMV infection and induction of EH in the Kazakh population. Unfortunately, the experimental results did not validate our hypothesis. The most likely explanation for this is the influence of environmental factors on epigenetics, suggesting that genetic background, lifestyle, and environmental exposure in Kazakhs may predispose these individuals to EH.

The present study had several limitations. First, the small sample size prevented a deeper analysis of the relationship between HCMV infection and DNA methylation. In addition, we were unable to isolate RNA for our epigenetic analysis as a result of the poor environmental conditions during blood sample collection. For these reasons, our findings should be interpreted with caution and should be examined in further studies.

In summary, our study showed that lower *ACE* and increased *HSD3B2* methylation play an important pathological role underlying HCMV-induced EH. Thus, the association between HCMV and clinical laboratory values support that increased TNF-α and 8-OHDG and decreased eNOS and renin may facilitate EH development. Moreover, our results suggest that infection with virus represents a risk factor for unfavorable changes in the cardiovascular system in the Kazakh population. Ultimately, to further confirm our findings, we must do additional studies on the development mechanism of hypertension in Kazakhs.

## Abbreviations

8-OHDG: 8-hydroxy-2-deoxyguanosine
*ACE*: angiotensin-converting enzyme
*ADD1*: α-adducin 1
Ang II: angiotensin II
AR: adrenergic receptors
BMI: body mass index
BP: blood pressure
CMV: cytomegalovirus
CpG: cytosine-phosphate-guanine dinucleotide
CRP: C-reactive protein
CVD: cardiovascular disease
DBP: diastolic blood pressure
EH: essential hypertension
eNOS: endothelial nitric oxide synthase
FBS: fasting blood sugar
HCMV: human cytomegalovirus
HDL-C: serum high-density lipoprotein cholesterol
HSD11β2; *HSD3B2*: 11-β hydroxysteroid dehydrogenase 2
LDL-C: serum low-density lipoprotein cholesterol
MAP: mean arterial pressure
NO: nitric oxide
NOX4: NADPH oxidase 4
RAS: renin–angiotensin system
ROS: reactive oxygen species
sACE: serum angiotensin-converting enzyme
SBP: systolic blood pressure
*SULF1*: sulphatase 1
TC: total cholesterol
TG: triglyceride
TNF-α: tumor necrosis factor-α
WHR: waist-hip ratio
